# Internet-Based Early Intervention to Prevent Posttraumatic Stress Disorder in Injury Patients: Randomized Controlled Trial

**DOI:** 10.2196/jmir.2460

**Published:** 2013-08-13

**Authors:** Joanne Mouthaan, Marit Sijbrandij, Giel-Jan de Vries, Johannes B Reitsma, Rens van de Schoot, J Carel Goslings, Jan SK Luitse, Fred C Bakker, Berthold PR Gersons, Miranda Olff

**Affiliations:** ^1^Center for Anxiety Disorders, Research Group PsychotraumaDepartment of PsychiatryAcademic Medical CenterAmsterdamNetherlands; ^2^Department of Clinical PsychologyVU UniversityAmsterdamNetherlands; ^3^EMGO Institute for Health and Care ResearchAmsterdamNetherlands; ^4^Department of Clinical Epidemiology, Biostatistics and BioinformaticsAcademic Medical CenterAmsterdamNetherlands; ^5^Julius Center for Health Sciences and Primary CareUniversity Medical CenterUtrechtNetherlands; ^6^Department of Methodology and StatisticsUtrecht UniversityUtrechtNetherlands; ^7^Optentia Research ProgramFaculty of HumanitiesNorth-West UniversityPotchefstroomSouth Africa; ^8^Trauma UnitDepartment of SurgeryAcademic Medical CenterAmsterdamNetherlands; ^9^Department of TraumatologyVU University Medical CenterAmsterdamNetherlands; ^10^Arq Psychotrauma Expert GroupDiemenNetherlands

**Keywords:** early intervention, prevention, Internet, posttraumatic stress disorder, cognitive behavior therapy

## Abstract

**Background:**

Posttraumatic stress disorder (PTSD) develops in 10-20% of injury patients. We developed a novel, self-guided Internet-based intervention (called Trauma TIPS) based on techniques from cognitive behavioral therapy (CBT) to prevent the onset of PTSD symptoms.

**Objective:**

To determine whether Trauma TIPS is effective in preventing the onset of PTSD symptoms in injury patients.

**Methods:**

Adult, level 1 trauma center patients were randomly assigned to receive the fully automated Trauma TIPS Internet intervention (n=151) or to receive no early intervention (n=149). Trauma TIPS consisted of psychoeducation, in vivo exposure, and stress management techniques. Both groups were free to use care as usual (nonprotocolized talks with hospital staff). PTSD symptom severity was assessed at 1, 3, 6, and 12 months post injury with a clinical interview (Clinician-Administered PTSD Scale) by blinded trained interviewers and self-report instrument (Impact of Event Scale—Revised). Secondary outcomes were acute anxiety and arousal (assessed online), self-reported depressive and anxiety symptoms (Hospital Anxiety and Depression Scale), and mental health care utilization. Intervention usage was documented.

**Results:**

The mean number of intervention logins was 1.7, SD 2.5, median 1, interquartile range (IQR) 1-2. Thirty-four patients in the intervention group did not log in (22.5%), 63 (41.7%) logged in once, and 54 (35.8%) logged in multiple times (mean 3.6, SD 3.5, median 3, IQR 2-4). On clinician-assessed and self-reported PTSD symptoms, both the intervention and control group showed a significant decrease over time (*P*<.001) without significant differences in trend. PTSD at 12 months was diagnosed in 4.7% of controls and 4.4% of intervention group patients. There were no group differences on anxiety or depressive symptoms over time. Post hoc analyses using latent growth mixture modeling showed a significant decrease in PTSD symptoms in a subgroup of patients with severe initial symptoms (n=20) (*P*<.001).

**Conclusions:**

Our results do not support the efficacy of the Trauma TIPS Internet-based early intervention in the prevention of PTSD symptoms for an unselected population of injury patients. Moreover, uptake was relatively low since one-fifth of individuals did not log in to the intervention. Future research should therefore focus on innovative strategies to increase intervention usage, for example, adding gameplay, embedding it in a blended care context, and targeting high-risk individuals who are more likely to benefit from the intervention.

**Trial Registration:**

International Standard Randomized Controlled Trial Number (ISRCTN): 57754429; http://www.controlled-trials.com/ISRCTN57754429 (Archived by WebCite at http://webcitation.org/6FeJtJJyD).

## Introduction

Posttraumatic stress disorder (PTSD) develops after trauma exposure, such as violence, disasters, and injury [1,2]. PTSD’s lifetime prevalence in adults is 7-8% [3,4], whereas the conditional prevalence rate after exposure to violence or injury ranges from 10-56% [1,3,5]. PTSD symptoms include intrusions of the traumatic event, avoidance of stimuli related to the event, emotional numbness, and hyperarousal [6]. Until now, efforts to prevent PTSD onset, for example, psychological debriefing, have been unsuccessful [7,8]. Early treatment of PTSD, or its precursor Acute Stress Disorder, with 4-5 sessions of trauma-focused cognitive behavioral therapy (CBT) was found to be effective in preventing chronic PTSD [9]. CBT consists of imaginal exposure to the traumatic incident, aimed at extinction of the original fear associations [10], and stress-management techniques and cognitive restructuring to correct irrational beliefs [11]. A recent randomized controlled trial found evidence for the effectiveness of 3 sessions of prolonged (imaginal) exposure, starting within 12 hours of the traumatic event, in counteracting later symptoms of PTSD and depression [12]. It is yet unclear whether CBT-techniques administered as a single session early intervention are effective in preventing PTSD.

We developed Trauma TIPS, a brief self-guided Internet intervention based on established CBT techniques. Trauma TIPS aims to decrease acute levels of distress, anxiety, and arousal, known to predict PTSD [13], and to prevent the onset of PTSD symptoms by providing information on successful coping, instructions for self-exposure to fearful situations, and stress management techniques. The exponential growth of global Internet use contributes to the feasibility of e-mental health interventions, which are considered a cost-effective alternative to traditional interventions [14]. Although both self-guided and therapist-assisted Internet-based CBT programs have been successful in the *treatment* of PTSD [15], there is a great lack of study into whether these programs may *prevent* PTSD. Preliminary evidence from one previous study on the efficacy of a self-guided Internet-based psychoeducational program for injured children and their parents showed greater anxiety reductions in children who had completed the program compared to those who had not [16].

Our study examined whether Trauma TIPS prevents the onset of PTSD symptoms in injury patients compared to care as usual. In addition, we evaluated whether Trauma TIPS prevented symptoms of depression and anxiety and led to a decrease in mental health care utilization during the first year after injury.

## Methods

### Trial Design

This study was an assessor-blinded randomized controlled trial (RCT; ISRCTN57754429) comparing a brief Internet-based early psychological intervention with a care-as-usual control group in two trauma centers (see Multimedia Appendix 1 for the CONSORT E-HEALTH Checklist of the trial).

### Participants

Injury patients transported by ambulance or helicopter to the level 1 trauma centers of the Academic Medical Center (AMC) and VU University Medical Center (VUmc) in Amsterdam, the Netherlands, were eligible for inclusion. These patients were suspected to suffer from possible severe injuries that required specialized acute medical care. Inclusion criteria were age 18 years or older, proficiency in Dutch, and having experienced a potential traumatic event (cf. Criterion A1 DSM-IV PTSD diagnosis) [6]. According to this criterion, the person has experienced, witnessed, or been confronted with an event or events that involve actual or threatened death or serious injury, or a threat to the physical integrity of oneself or others. Exclusion criteria were the injury resulting from deliberate self-harm ; organic brain condition, psychotic disorder, bipolar disorder, or depression with psychotic features (cf. DSM-IV) [6]; moderate to severe traumatic brain injury (TBI) (according to a Glasgow Coma Score [17] less than 13); and permanent residency outside the Netherlands.

### Interventions

Trauma TIPS [18] (for screenshots see Multimedia Appendices 2 and 3) was created and is owned by the authors from the Research Group Psychotrauma [19]. It is based on CBT techniques of psychoeducation, stress management/relaxation techniques, and in vivo exposure. It consists of 6 steps, including introduction to the program and basic operating instructions; assessments of acute anxiety and arousal using Visual Analogue Scales (VAS) at pre- and postintervention; video features of the trauma center’s surgical head explaining the procedures at the center and the purpose of the program, and of 3 patient models sharing their experiences after their injury; a short textual summary of 5 coping tips for common physical and psychological reactions after trauma; audio clips with instructions for stress management techniques; contact information for program assistance or professional help for enduring symptoms; and a Web forum for peer support. The introduction page shows the logos of the academic hospitals involved in the study, as well as the logos of the funders of the study. The full design and content of the intervention are described elsewhere [19,20]. Total duration of the program was approximately 30 minutes. Care as usual, available to patients from both groups, consisted of incidental, nonstructured talks with trauma center staff or with a patient’s general practitioner (GP), either directly following injury or during the course of the trial.

### Study Procedures

The local institutional review boards provided medical ethical approval. Patients were contacted in hospital or via telephone within 72 hours post injury to assess eligibility and to schedule a baseline assessment. Informed consent was obtained face-to-face directly prior to the baseline assessment at approximately 1 week post injury. Patients were randomly allocated to (1) the Trauma TIPS intervention or (2) a control group with no intervention, but access to care as usual. Randomization was performed by a research member independent of data collection in a 1:1 ratio by a computerized program, TENALEA Clinical Trial Data Management System (NKI/AVL Biometrics department, Amsterdam), using random block sizes (with maximum block size 6), stratified by study center. Intervention group patients received personal log-in codes for the intervention’s website, along with instructions to perform the intervention at will, but at least once within the first month. Electronic and telephone reminders were sent to encourage (early) log-in, but patients were free to access the intervention as they pleased, to underscore the intervention’s voluntary nature and self-guiding principles. Research assistants visited patients with a laptop in case of hospitalization or a lack of Internet or computer access. Follow-up assessments were scheduled at 1, 3, 6, and 12 months post injury. The assessments took place at the AMC’s Center for Anxiety Disorders, at bedside in the hospital, or at the private home of the patient. Patients were asked not to share information about the randomization to the assessors, to ensure that they were blind to the allocated interventions. No reimbursement was given.

### Outcomes

Trained assessors at the master’s and doctoral levels performed the data collection. The main outcome measure was PTSD symptom severity on the Clinician-Administered PTSD Scale (CAPS) [21]. The structured interview assesses the frequency and intensity (ranging from 0-4) of the 17 DSM IV symptoms of PTSD (total scores range from 0-136). Scores are added to represent PTSD symptom severity or a diagnosis. The internal consistency of the Dutch translation of the CAPS is good to excellent [22]. Presence of a PTSD diagnosis was computed using the established rule of Weathers et al [23].

The Mini International Neuropsychiatric Interview (MINI-Plus, version 5.0) [24], a semistructured clinical interview, was used to obtain DSM IV diagnoses of major depressive disorders (MDD) and anxiety disorders other than PTSD. Each module starts with screening questions, which, if positive, lead to a further examination of the disorder’s criteria.

We assessed self-reported PTSD severity with the Impact of Event Scale-Revised (IES-R) [25]. The 22 items are scored on a 5-point scale, from 0 (not at all) to 4 (extremely). Total scores range from 0-88 with higher scores representing more severe symptoms. The IES-R shows high internal consistency [25,26].

Self-reported severity of depressive and anxiety symptoms was assessed using the Hospital Anxiety and Depression Scale (HADS) [27]. The item scores in the two subscales of depression (7 items) and anxiety (7 items) range from 0-3 (total scores per subscale ranging from 0-21). Higher scores indicate greater symptomatology. The test-retest reliability of the 2 scales is high [28].

The Trimbos/iMTA questionnaire for Costs associated with Psychiatric illness (TiC-P) [29] was used to evaluate direct and indirect health costs. Direct costs include contacts with mental health professionals (eg, GP, psychologist, social worker), medication use, and admissions for mental health problems. Indirect costs were calculated as production losses due to psychological problems by the Short Form Health and Labour Questionnaire (SF-HLQ) [30].

At the beginning and after completion of Trauma TIPS, patients indicated acute anxiety and arousal levels from 0 (no anxiety or arousal) to 100 (worst anxiety or arousal) on two online VASs [19,20].

Website activity was recorded to evaluate usage characteristics, such as number of log-ins and total amount of login time.

### Sample Size

To demonstrate a difference of at least 5.5 points on the CAPS between the groups at 12 months, equivalent to a small to medium effect size of Cohen’s *d*=.35, 134 patients or more per condition were required (Cronbach alpha=.05, power=80%, SD 16) [31]. Anticipating possible attrition of study participants, we included 150 patients per condition.

### Analyses

Differences in baseline characteristics between the study groups, patients lost to follow-up vs patients not lost to follow-up and patient groups with varying intervention usage were tested using independent sample *t* tests and chi-square tests (Bonferroni adjusted *P*=.005). Missing data were imputed using general purpose multivariate imputation procedure (ICE: sequential regression imputation method), creating 50 different datasets. All analyses were performed using these 50 datasets and then pooled by combining the individual results. Due to their positive skewness, CAPS and IES-R values were square root transformed. Stata version 11.2 was used for all repeated measures analyses of PTSD symptoms (CAPS, IES-R) and depressive and anxiety symptoms (HADS-A, HADS-D). The effects of time of measurement, group, and the group-by-time interaction were analyzed with linear mixed models. For all regression models, a robust variance estimator was used. Estimated values (adjusted) and 95% confidence intervals (CIs) are presented throughout the paper unless otherwise specified. Finally, as a post hoc analysis, we applied latent growth mixture modelling (LGMM) [32,33] to explore possible latent subgroups within the two groups by use of the software Mplus (Version 6.11) [34] using a Bayesian estimator [35,36]. Across all analyses, two-tailed tests are reported with Cronbach alpha=.05.

## Results

### Baseline Characteristics

**Figure 1:**
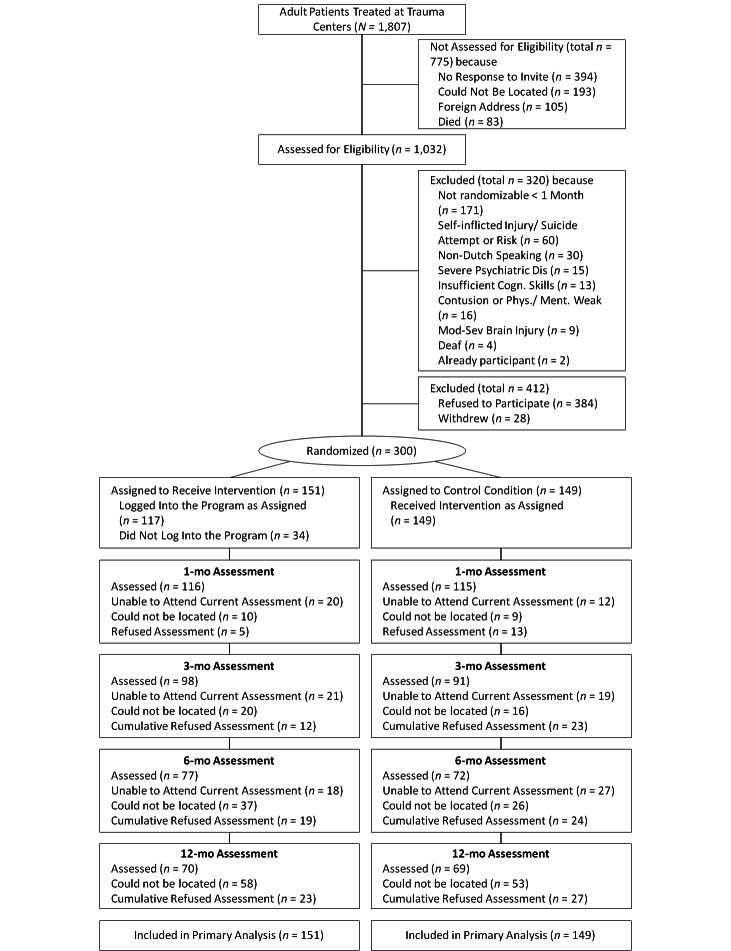
Flow of participants through the trial.

Recruitment and follow-up took place from September 2007 to June 2010. Figure 1 shows the flow of patients through the trial. Participants were significantly older (mean age 43.8, SD 15.9) than patients who refused participation (mean age 40.1, SD 16.3, *P*=.01). Table 1 shows the baseline characteristics of participants. There were no differences in baseline characteristics or attrition rate between the study groups. Patients lost to the 12-month follow-up were more often unmarried than patients who were not lost to follow-up (*P*=.001).

### Intervention Usage

Most intervention group patients logged in to the intervention’s website once (n=63, 41.7%). Fifty-four patients (35.8%) logged in multiple times (mean 3.6, SD 3.5, median 3, IQR 2-4). Thirty-four patients (22.5%) did not log in (ie, nonusers) and provided the following reasons: not interested anymore (2), occupied with rehabilitation (1), too busy (1), on holiday (1), too much on my mind (1), tired (1), difficulty concentrating (1), postconcussion symptoms (1), broken back (1), husband deceased (1), or no explanation (22). The average number of log-ins for the entire group was 1.7 (SD 2.5). The average login time was 20.8 minutes (SD 26.3). There were no differences in attrition or outcome measures between nonusers (n=34) and users of the intervention (n=117), or between patients with a single log-in (n=63) versus multiple log-ins (n=54). The only differences were that more nonusers than users had a non-Dutch cultural background (*P*=.003) and that patients with multiple log-ins were significantly older (mean age 48.0, SD 14.6) than those with a single log-in (mean age 39.6, SD 14.1, *P*=.001).

From pre- to postintervention, the majority of intervention group patients reported no change in acute anxiety (55.9%, n=38) and arousal (63.2%, n=43) on the VASs. Seven patients reported an increase (10.3%), and 23 (33.8%) and 18 (26.5%) patients reported a reduction in anxiety and arousal respectively.

### Main Outcomes

**Figure 2:**
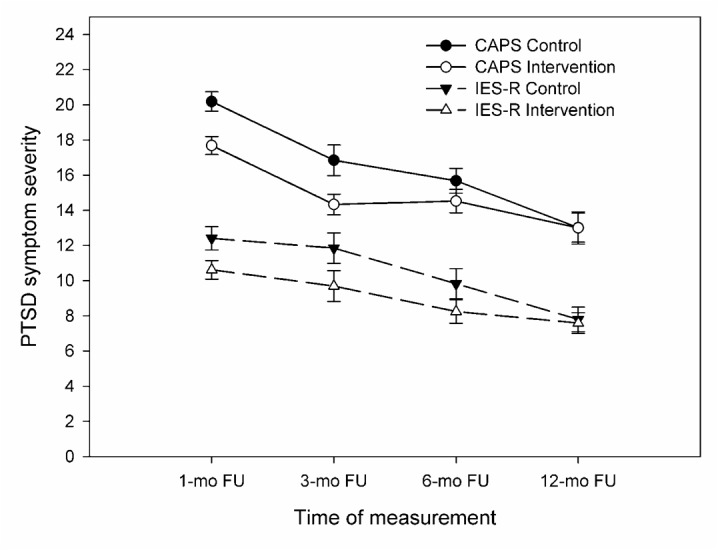
Trends in observed PTSD symptom severity (CAPS and IES-R) per intervention group.

Table 2 shows the results of the intention-to-treat analyses for PTSD, anxiety, and depressive symptoms. Mixed-model analysis of PTSD symptom severity of the CAPS showed a significant effect of time (*P*<.001), but no significant group differences over time (12-month follow-up, Internet intervention group: estimated means 13.0, 95% CI 11.2 - 14.8; control group: estimated means 13.0, 95% CI 11.4 - 14.6, *P*=.63). On the mixed-model analysis of self-reported PTSD symptoms (IES-R), we found a similar significant time effect (*P*<.001) and no group differences over time (12-month follow-up, Internet intervention group: estimated means 7.6, 95% CI 6.4 - 8.7; control group: estimated means 7.8, 95% CI 6.4 - 9.2, *P*=.76). Figure 2 presents the estimated CAPS and IES-R means over time. For depressive and anxiety symptoms, we found no effects of time or group over time in mixed-model analyses (12 month HADS-D, Internet intervention group: estimated means 3.3, 95% CI 2.4 - 4.2; control group: estimated means 3.0, 95% CI 2.2 - 3.7, *P*=.72; 12 month HADS-A, Internet intervention group: estimated means 4.1, 95% CI 3.5 - 4.8; control group: estimated means 3.7, 95% CI 3.0 - 4.3, *P*=.53).

PTSD was diagnosed in 9.2% of patients at 1 month (n=21), 7.6% at 3 months (n=14), 7.5% at 6 months (n=11), and 4.5% at 12 months (n=6). MDD was diagnosed in 7.6% of patients at 1 month (n=17), 2.7% at 3 months (n=5), 7.6% at 6 months (n=11), and 6.8% at 12 months (n=9). Ten patients (4.4%) were diagnosed with an anxiety disorder at 1 month, 11 patients (6.0%) at 3 months, 14 patients (9.7%) at 6 months, and 10 patients (7.6%) at 12 months. chi-square analyses showed no group differences in prevalence of any of the psychiatric diagnoses.

Mental health care utilization at 12 months was similar for both groups, such as visits to a GP (*P=*.35), company doctor (*P=*.95), mental health specialists (*P=*.52), hospital admissions (*P=*.70), or medication use (*P=*.57).The groups also did not differ with respect to employment status (*P=*.70), working hours (*P=*.89), and work absence (**P*=.*81). Due to the absence of significant group differences, the direct and indirect costs for mental health use were not calculated.

### Completer Analyses

In completers-only analyses (n=117 intervention group and n=149 control group patients), excluding nonusers (n=34), results were similar to the intention-to-treat results for all outcome measures.

### Latent Subgroups

Post hoc LGMM analyses of self-reported PTSD symptoms (IES-R) revealed two latent subgroups per study group based on PTSD symptom severity at baseline, resulting in a low symptomatic control subgroup (n=94) and intervention subgroup (n=105), and a high symptomatic control subgroup (n=15) and intervention subgroup (n=20). The main difference between the groups was the slope of the high symptomatic subgroups, which showed a significant decrease in the intervention subgroup (*P*<.001), but not in the control subgroup (*P=*.32). Table 3 shows the outcomes of the LGMM analyses.

**Table 1:** Participant characteristics at baseline.

Characteristic		Internet intervention n=151	Control with usual care n=149	*P* value^a^
Age in years, mean (SD)		44.18 (15.76)	43.49 (16.00)	.54
Sex (male), n (%)		89 (58.9)	91 (61.1)	.73
Post-high school education, n (%)		37 (24.7)	43 (29.1)	.71
Unemployed, n (%)		41 (27.5)	29 (19.5)	.13
Married/cohabitating, n (%)		82 (54.3)	81 (54.4)	.54
Dutch cultural background, n (%)		127 (84.1)	122 (83.0)	.88
Prior traumatic events, mean (SD)		2.99 (2.42)	2.93 (2.20)	.80
Hospital admission, n (%)		100 (66.7)	105 (70.9)	.46
Days hospitalized, mean (SD)		5.30 (8.02)	4.57 (7.36)	.20
ICU admission, n (%)		13 (8.7)	13 (8.8)	.97
Injury Severity Score, mean (SD)		10.45 (8.59)	10.21 (9.87)	.33
Glasgow Coma Scale, mean (SD)		14.48 (1.91)	14.72 (1.42)	.08
**Traumatic event, n (%)**				.11
	Traffic accident	99 (65.6)	105 (70.5)	
	Work-related accident	12 (7.9)	16 (10.7)	
	Fall	28 (18.5)	13 (8.7)	
	Interpersonal violence/physical abuse	2 (2.3)	5 (3.4)	
	Other	10 (6.6)	10 (6.7)	
**Psychological assessment tools, mean (SD)**				
	Impact of Event Scale—Revised	17.60 (16.82)	21.22 (19.09)	.15
	Hospital Anxiety and Depression Scale—Depression	3.69 (3.50)	4.13 (4.26)	.09
	Hospital Anxiety and Depression Scale—Anxiety	4.36 (3.90)	4.87 (4.33)	.21

^a^Independent *t* test for difference between groups for continuous measures and chi-square test for differences between groups in categorical characteristics.

**Table 2:** Outcomes of intention-to-treat linear mixed models for PTSD, depressive, and anxiety symptoms.^a^

Outcome		Internet intervention	Control with usual care n=149	Time		Group		Group x Time	
				*F*	*P*	*F*	*P*	*F*	*P*
**Clinician-assessed PTSD symptoms (CAPS)**				6.3	<.001	1.7	.19	0.6	.63
	1 month follow-up	17.7 (16.7 to 18.7)	20.2 (19.1 to 21.3)						
	3 month follow-up	14.3 (13.2 to 15.5)	16.8 (15.1 to 18.6)						
	6 month follow-up	14.5 (13.2 to 15.8)	15.7 (14.3 to 17.1)						
	12 month follow-up	13.0 (11.2 to 14.8)	13.0 (11.4 to 14.6)						
**Patient-reported PTSD symptoms (IES-R)**				15.7	<.001	1.9	.17	0.5	.76
	1 month follow-up	10.6 (9.6 to 11.7)	12.4 (11.1 to 13.7)						
	3 month follow-up	9.7 (8.0 to 11.4)	11.8 (10.1 to 13.5)						
	6 month follow-up	8.2 (6.9 to 9.6)	9.8 (8.1 to 11.5)						
	12 month follow-up	7.6 (6.4 to 8.7)	7.8 (6.4 to 9.2)						
**Anxiety symptoms (HADS-A)**				2.2	.07	0.3	.57	0.8	.53
	1 month follow-up	4.6 (3.9 to 5.2)	4.8 (4.1 to 5.5)						
	3 month follow-up	4.0 (3.5 to 4.5)	4.3 (3.8 to 4.9)						
	6 month follow-up	3.9 (3.2 to 4.6)	4.6 (3.7 to 5.4)						
	12 month follow-up	4.1 (3.4 to 4.8)	3.7 (3.0 to 4.3)						
**Depressive symptoms (HADS-D)**				2.3	.054	0.3	.62	0.5	.72
	1 month follow-up	3.6 (3.2 to 4.0)	4.1 (3.5 to 4.6)						
	3 month follow-up	3.5 (3.0 to 4.0)	3.9 (3.4 to 4.5)						
	6 month follow-up	4.1 (3.5 to 4.8)	4.5 (3.6 to 5.4)						
	12 month follow-up	3.3 (2.4 to 4.2)	3.0 (2.2 to 3.7)						

^a^Data are expressed as mean (95% CI).

**Table 3:** Outcomes of latent growth mixture modeling analyses for self-reported PTSD severity (IES-R).

Latent subgroups		Internet intervention			Control with usual care		
		n	mean (95% CI)	*P*	n	mean (95% CI)	*P*
**Low symptomatic subgroup**		105			94		
	Intercept		9.0 (6.9 to 11.1)	<.001		14.9. (11.4 to 18.5)	<.001
	Slope		-1.0 (-1.4 to -0.4)	<.001		-1.4 (-1.9 to -0.8)	<.001
**High symptomatic subgroup**		20			15		
	Intercept		41.2 (35.0 to 50.3)	<.001		42.9 (30.1 to 55.6)	<.001
	Slope		-3.6 (-5.2 to -2.1)	<.001		0.6 (-2.7 to 3.9)	.32

## Discussion

### Principal Findings

In this paper, we presented the results of a randomized clinical trial comparing a self-guided Internet-based prevention program vs usual care in the prevention of PTSD symptoms in injury patients. PTSD symptoms decreased over time without a significant difference between the Internet intervention group and the control group. Moreover, there were no differences between groups with respect to the number of PTSD and MDD diagnoses and with respect to the severity of depression and anxiety at 12 months. An important finding is that participants were reluctant to use the intervention. In fact, one in five patients in the intervention group lacked any exposure to the intervention. Based on these results, there are currently no indications that offering a voluntary, information-based prevention program via the Internet to unselected injury victims is useful in preventing PTSD symptoms.

The low adherence rates were comparable to those found in similar self-help Internet-based interventions [37]. In part, this nonusage was a consequence of a deliberate design choice to allow patients freedom in performing the intervention, having learned from adverse effects of debriefing interventions found previously to be noneffective or even harmful [7,13]. However, in order to induce changes in behavior and affect, true exposure to an intervention is necessary, which entails accessing the intervention website, staying on the intervention website to actually use it, and revisiting the intervention website, in case of a repetitive design [38]. As possible reasons for dropping out of or not adhering to online treatment programs, previous studies reported time constraints, lack of motivation, technical or computer-access problems, depressive episode or physical illness, the lack of face-to-face contact, a preference for taking medication, perceived lack of treatment effectiveness, improvement in condition, and burden of the program [37]. Strategies to increase uptake of Trauma TIPS may be a more structured peer-support forum, more interactive elements to the intervention, such as quizzes or knowledge questions, automated feedback on the acute anxiety and arousal assessments, or monetary incentive [38-40]. Moreover, a more strict approach to intervention adherence for inclusion in our study (eg, a minimum number of log-ins or log-in time required for participation) may have resulted in greater benefits. However, note that we found no differences in outcomes between users and nonusers or between participants with single versus multiple use. Finally, it is possible that the idea of a computerized program did not match the acute needs of the injury victims, resulting in some of them not using it. Previous studies investigating needs of victims after the September 11, 2001, terrorist attacks and the 2005 London bombings showed that only very few people (< 1%) reported a need for professional mental health support in the acute posttrauma phase, and most (71-87%) turned to loved ones or others for support [41,42].

Another explanation for not finding a significant effect of the intervention may be the low overall PTSD symptom level. Only 9.2% of patients developed PTSD at 1 month, which decreased to 4.5% at 12 months. Beforehand, we expected that 19% of participants would have developed PTSD at 3 months [31]. This unexpectedly low PTSD incidence left little room for symptom improvement for the whole group. Additionally, the relatively low symptom levels may have caused participants to experience little personal incentive to access and use the intervention. Support for this comes from our post hoc subgroup analyses that suggested that the Trauma TIPS intervention was effective in reducing PTSD symptoms in individuals with high initial symptom levels. Because this subgroup was small (n=20), these results must be interpreted with caution.

Internet interventions may not be suitable for all individuals. Common points of criticism are that the mainly information-driven formats pose a disadvantage to people with lesser reading or language skills, do not meet the needs of the elderly or persons with limited computer skills or experience, and that it is difficult to appeal to a culturally diverse audience in a single format, as possibly illustrated in our sample of more nonusers having a non-Dutch cultural background [37]. On the other hand, the rapid developments in Internet applications, especially via mobile technology, provide more possibilities to reach populations who were earlier underserved in eHealth care [43,44].

### Limitations

One limitation of our study was missing data due to patient dropout or failure to complete self-report instruments. We do not know to what extent attrition may have biased our results, although besides marital status, we found no differences between participants and dropouts. In addition, our sample may not have been fully representative of the entire level 1 trauma center population, since we excluded patients with moderate-severe TBI, who did not master the Dutch language, or who were unable to meet our time requirements for logging in.

### Conclusions

As a clinical implication of our study, future comparable Internet-based early interventions should be aimed at individuals with high initial symptoms. These individuals may be accurately identified within the first weeks following trauma with early screening tools for PTSD [45-47]. Stepped care programs for acutely traumatized individuals have recently shown to be feasible [48]. The results of our study show that an e-mental health approach could well be a first step in the acute aftercare of highly distressed trauma victims, since Trauma TIPS was indeed effective in a latent subgroup of participants experiencing high levels of PTSD symptoms at baseline. For those victims whose symptoms remain, our self-guided early intervention could be followed by more specialized or traditional curative face-to-face treatment as part of a blended care strategy [49].

Future studies may determine the effectiveness of applying interventions such as Trauma TIPS to individuals with high levels of distress. They may also evaluate whether incorporation of strategies to increase adherence, for instance a motivating interviewing module or increasing the fun by adding serious gaming components to Trauma TIPS, may increase its effectiveness.

In conclusion, our study found no evidence for preventing the development of PTSD symptoms by offering a voluntary, information-based prevention program via the Internet to unselected injury trauma victims. Future research may focus on innovative strategies to increase intervention usage and targeting high-risk individuals who are more likely to benefit from the intervention.

## Multimedia Appendix 1

CONSORT-EHEALTH checklist V1.6.2 [42].

## Multimedia Appendix 2

Screenshot of the Trauma TIPS internet intervention: video feature of the trauma center’s surgical head.

## Multimedia Appendix 3

Screenshot of the Trauma TIPS internet intervention: video features of patient models.

## Abbreviations

AMC: Academic Medical Center
CAPS: Clinician Administered PTSD Scale
CBT: cognitive behavioral therapy
GP: general practitioner
HADS: Hospital Anxiety and Depression Scale
HADS-A: Hospital Anxiety and Depression Scale-Anxiety subscale
HADS-D: Hospital Anxiety and Depression Scale-Depression subscale
IES-R: Impact of Event Scale-Revised
IQR: interquartile range
LGMM: latent growth mixture modeling
MDD: major depressive disorder
MINI-Plus: Mini International Neuropsychiatric Interview
PTSD: posttraumatic stress disorder
RCT: randomized controlled trial
SF-HLQ: Short Form Health and Labour Questionnaire
TBI: traumatic brain injury
TiC-P: Trimbos/iMTA Questionnaire for Costs Associated with Psychiatric Illness
VAS: Visual Analogue Scale
VUmc: VU University Medical Center
