# Supplementary material for: Internet-Based Early Intervention to Prevent Posttraumatic Stress Disorder in Injury Patients: Randomized Controlled Trial
Source: J Med Internet Res. 2013 Aug 13;15(8):e165. doi: 10.2196/jmir.2460 (PMC3742408; doi:10.2196/jmir.2460)
Supplement: Supplementary file 1 [file jmir_v15i8e165_app1.pdf]

CONSORT-EHEALTH Checklist V1.6.2 Report

(based on CONSORT-EHEALTH V1.6), available at [http://tinyurl.com/consort-ehealth-v1-6].

2460

Date completed

11/28/2012 9:39:28

by

Joanne Mouthaan

Internet-based Early Intervention to Prevent Posttraumatic Stress Disorder in Injury Patients: A Randomized Controlled Trial

TITLE

1a-i) Identify the mode of delivery in the title

Title: Internet-based Early Intervention to Prevent Posttraumatic Stress Disorder in Injury Patients: A Randomized Controlled Trial

1a-ii) Non-web-based components or important co-interventions in title

No, control group does not receive any alternative intervention, only care as usual.

1a-iii) Primary condition or target group in the title

Title: Internet-based Early Intervention to Prevent Posttraumatic Stress Disorder in Injury Patients: A Randomized Controlled Trial

ABSTRACT

1b-i) Key features/functionalities/components of the intervention and comparator in the METHODS section of the ABSTRACT

The abstract includes these phrases:
- "a novel, self-guided internet-based intervention (called Trauma TIPS) based on techniques from cognitive behavioral therapy (CBT)"
- "internet intervention (n=151) or to no early intervention (n=149)"
- "Trauma TIPS consisted of psychoeducation, in vivo exposure and stress management techniques"

1b-ii) Level of human involvement in the METHODS section of the ABSTRACT

Abstract includes phrase: "randomly assigned to receive the fully automated Trauma TIPS internet intervention (n=151)"

1b-iii) Open vs. closed, web-based (self-assessment) vs. face-to-face assessments in the METHODS section of the ABSTRACT

"PTSD symptom severity was assessed at 1, 3, 6 and 12 months post-injury using the Clinician Administered PTSD Scale by blinded trained interviewers and self-report instrument (Impact of Events Scale-Revised). Secondary outcomes were acute anxiety and arousal (assessed online), self-reported depressive and anxiety symptoms and mental health care utilization."

1b-iv) RESULTS section in abstract must contain use data

"Results. The mean number of intervention logins was 1.7 (SD=2.5, Mdn=1, IQR=1-2). Thirty-four patients did not log in (22.5%), 63 (41.7%) logged in once and 54 (35.8%) logged in multiple times (M=3.6, SD=3.5, Mdn=3, IQR=2-4). On clinician assessed and self-reported PTSD symptoms, both the intervention and control group showed a significant decrease over time (P<.001) without significant differences in trend. PTSD at 12 months was diagnosed in 4.7% of controls and 4.4% of intervention group patients. There were no group differences on anxiety or depressive symptoms over time."

1b-v) CONCLUSIONS/DISCUSSION in abstract for negative trials

"Conclusions. Our results do not support the efficacy of the Trauma TIPS internet-based early intervention in the prevention of PTSD symptoms for an unselected population of injury patients. Future research should point out the efficacy of applying interventions to selected individuals and should focus on increasing intervention usage."

INTRODUCTION

2a-i) Problem and the type of system/solution

"Until now, efforts to prevent PTSD onset, e.g. psychological debriefing, have been unsuccessful [7,8]. [...]. It is yet unclear whether CBT-techniques administered as a single session early intervention are efficacious in preventing PTSD. We developed Trauma TIPS, a brief self-guided internet intervention based on established CBT-techniques."

2a-ii) Scientific background, rationale: What is known about the (type of) system

"Although both self-guided and therapist-assisted internet-based CBT programs have been successful in the treatment of PTSD [16], there is a great lack of studies that tested whether these programs may prevent PTSD. Our study examined whether Trauma TIPS prevents the onset of PTSD symptoms in injury patients compared to care as usual. In addition, we evaluated whether Trauma TIPS prevented symptoms of depression and anxiety and led to a decrease in mental health care utilization during the first year after injury."

METHODS

3a) CONSORT: Description of trial design (such as parallel, factorial) including allocation ratio

"Trauma TIPS aims to decrease acute levels of distress, anxiety and arousal, known to predict PTSD [13], and to prevent the onset of PTSD symptoms by providing information on successful coping, instructions for self-exposure to fearful situations and stress management techniques. [...] Our study examined whether Trauma TIPS prevents the onset of PTSD symptoms in injury patients compared to care as usual. In addition, we evaluated whether Trauma TIPS prevented symptoms of depression and anxiety and led to a decrease in mental health care utilization during the first year after injury."

3b) CONSORT: Important changes to methods after trial commencement (such as eligibility criteria), with reasons

NA

3b-i) Bug fixes, Downtimes, Content Changes

NA, no major changes made during trial.

4a) CONSORT: Eligibility criteria for participants

"Injury patients transported to the level-1 trauma centers of the Academic Medical Center (AMC) and VU University Medical Center (VUmc) Amsterdam, The Netherlands, were eligible for inclusion. Inclusion criteria were: having experienced a traumatic event (cf. Criterion A1 DSM-IV PTSD diagnosis) [6]; age 18 years or older; and proficiency in Dutch. Exclusion criteria were: the injury resulted from deliberate self-harm; organic brain condition, psychotic disorder, bipolar disorder or depression with psychotic features (cf. DSM-IV) [6]; moderate to severe traumatic brain injury (TBI) (according to a Glasgow Coma Score (GCS) [18] less than 13); and permanent residency outside the Netherlands."

4a-i) Computer / Internet literacy

"Research assistants visited patients with a laptop in case of hospitalization or a lack of internet or computer access."

4a-ii) Open vs. closed, web-based vs. face-to-face assessments:

"Patients were contacted in-hospital or via telephone within 72 hours post-injury to assess eligibility and to schedule a baseline assessment. [...] The assessments took place at the AMC's Center for Anxiety Disorders, at bedside in the hospital or at the private home of the patient."

4a-iii) Information giving during recruitment

Patients were informed about the specifics of the study face-to-face, since every assessment was performed during a clinical interview. Interviewers discussed what was expected from the participants in both conditions (in terms of intervention versus no intervention) and what the assesments entailed.

4b) CONSORT: Settings and locations where the data were collected

"The assessments took place at the AMC's Center for Anxiety Disorders, at bedside in the hospital or at the private home of the patient."

4b-i) Report if outcomes were (self-)assessed through online questionnaires
"At the beginning and after completion of Trauma TIPS, patients indicated acute anxiety and arousal levels from 0 (no anxiety or arousal) to 100 (worst anxiety or arousal) on 2 online VASs [19,20]."
4b-ii) Report how institutional affiliations are displayed
"The introduction page shows the logo's of the academic hospitals involved in the study, as well as the logo's of the funders of the study."
5) CONSORT: Describe the interventions for each group with sufficient details to allow replication, including how and when they were actually administered
5-i) Mention names, credential, affiliations of the developers, sponsors, and owners
"Trauma TIPS was created and is owned by the authors from the Research Group Psychotrauma [19]."
5-ii) Describe the history/development process
"The full design and content of the intervention are described elsewhere [19,20]."
5-iii) Revisions and updating
NA, no major revisions,updates or changes during trial.
5-iv) Quality assurance methods
NA. "The full design and content of the intervention are described elsewhere [19,20]."
5-v) Ensure replicability by publishing the source code, and/or providing screenshots/screen-capture video, and/or providing flowcharts of the algorithms used
Multimedia appendix 1
5-vi) Digital preservation
"Trauma TIPS (www.traumatips.nl, see Multimedia appendix 1)..."
5-vii) Access
"Intervention group patients received personal login codes for the intervention's website, along with instructions to perform the intervention at will, but at least once within the first month. Electronic and telephone reminders were sent to encourage (early) login, but patients were free to access the intervention as they pleased, to underscore the intervention's voluntary nature and self-guiding principles. Research assistants visited patients with a laptop in case of hospitalization or a lack of internet or computer access. [...] No reimbursement was given."
5-viii) Mode of delivery, features/functionalities/components of the intervention and comparator, and the theoretical framework
"Trauma TIPS [...] consists of 6 steps, including: introduction to the program and basic operating instructions; assessments of acute anxiety and arousal using Visual Analogue Scales (VASs) at pre- and post-intervention; video features of the trauma center's surgical head explaining the procedures at the center and the purpose of the program, and of 3 patient models sharing their experiences after their injury; a short textual summary of 5 coping tips for common physical and psychological reactions after trauma; audio clips with instructions for stress management techniques; contact information for program assistance or professional help for enduring symptoms; and a web forum for peer support. The introduction page shows the logo's of the academic hospitals involved in the study, as well as the logo's of the funders of the study. The full design and content of the intervention are described elsewhere [19,20]."
5-ix) Describe use parameters
"Website activity was recorded to evaluate usage characteristics, such as number of logins and total amount of login time."
5-x) Clarify the level of human involvement
NA, the intervention is fully automated and does not require input from others.
5-xi) Report any prompts/reminders used
"Electronic and telephone reminders were sent to encourage (early) login, but patients were free to access the intervention as they pleased, to underscore the intervention's voluntary nature and self-guiding principles."
5-xii) Describe any co-interventions (incl. training/support)
NA, control condition was care as usual (no intervention).
6a) CONSORT: Completely defined pre-specified primary and secondary outcome measures, including how and when they were assessed
"Outcomes
Trained master's- and doctoral level assessors, performed the data collection. The main outcome measure was PTSD symptom severity on the Clinician Administered PTSD Scale (CAPS) [21]. The structured interview assesses the frequency and intensity (ranging from 0-4) of the 17 DSM IV symptoms of PTSD (total scores range from 0-136). Scores are added to represent PTSD symptom severity or a diagnosis. The internal consistency of the Dutch translation of the CAPS is good to excellent [22]. Presence of a PTSD diagnosis was computed using the established rule of Weathers et al [23].
The Mini International Neuropsychiatric Interview (M.I.N.I.-Plus, version 5.0) [24], a semi-structured clinical interview, was used to obtain DSM IV diagnoses of major depressive disorder (MDD) and anxiety disorders other than PTSD,. Each module starts with screening questions which, if positive, lead to a further examination of the disorder's criteria.
We assessed self-reported PTSD severity with the Impact of Events Scale-Revised (IES-R) [25]. The 22 items are scored on a 5-point scale, from 0 (not at all) to 4 (extremely). Total scores range from 0-88 with higher scores representing more severe symptoms. The IES-R shows high internal consistency [25,26].
Self-reported severity of depressive and anxiety symptoms was assessed using the Hospital Anxiety and Depression Scale (HADS) [27]. The item scores in the 2 subscales depression (7 items) and anxiety (7 items) range from 0-3 (total scores per subscale ranging from 0-21). Higher scores indicate greater symptomatology. The test-retest reliability of the two scales is high [28].
The Trimbos/iMTA questionnaire for Costs associated with Psychiatric illness (TiC-P) [29] was used to evaluate direct and indirect health costs. Direct costs include contacts with mental health professionals (i.e., GP, psychologist, social worker), medication use and admissions for mental health problems. Indirect costs were calculated as production losses due to psychological problems by the Short Form Health and Labour Questionnaire (SF-HLQ) [30].
At the beginning and after completion of Trauma TIPS, patients indicated acute anxiety and arousal levels from 0 (no anxiety or arousal) to 100 (worst anxiety or arousal) on 2 online VASs [19,20].
Website activity was recorded to evaluate usage characteristics, such as number of logins and total amount of login time."
6a-i) Online questionnaires: describe if they were validated for online use and apply CHERRIES items to describe how the questionnaires were designed/deployed
Online assessments only included 2 VAS scales for anxiety and arousal, no questionnaires.
6a-ii) Describe whether and how "use" (including intensity of use/dosage) was defined/measured/monitored

"Intervention Usage
Most intervention group patients logged in on the intervention's website once (n=63, 41.7%). Fifty-four patients (35.8%) logged in multiple times (M=3.6, SD=3.5, Mdn=3, IQR=2-4). Thirty-four patients (22.5%) did not log in (e.g., non-users) and provided the following reasons: not interested anymore (2); occupied with rehabilitation (1); too busy (1); on holiday (1); too much on my mind (1); tired (1); difficulty concentrating (1); post-concussion symptoms (1); broken back (1); husband deceased (1); or no explanation (22). The average number of logins for the entire group was 1.7 (SD=2.5). The average login time was 20.8 minutes (SD=26.3). There were no differences in attrition or outcome measures between non-users (n=34) and users of the intervention (n=117), or between patients with a single login (n=63) versus multiple logins (n=54). The only differences were that more non-users than users had a non-Dutch cultural background (P=.003), and that patients with multiple logins were significantly older (mean age=48.0, SD=14.6) than those with a single login (mean age=39.6, SD=14.1; P=.001)."

6a-iii) Describe whether, how, and when qualitative feedback from participants was obtained

NA, pilot study results include qualitative feedback from participants and are published elsewhere.

6b) CONSORT: Any changes to trial outcomes after the trial commenced, with reasons

NA, no changes to trial outcomes.

7a) CONSORT: How sample size was determined

7a-i) Describe whether and how expected attrition was taken into account when calculating the sample size

"Sample size
To demonstrate a difference of at least 5.5 points on the CAPS between the groups at 12 months, equivalent to a small to medium effect size of Cohen's d=.35, 134 patients or more per condition were required (α=.05, power=80%, SD=16) [31]. Anticipating possible attrition of study participants, we included 150 patients per condition."

7b) CONSORT: When applicable, explanation of any interim analyses and stopping guidelines

NA, no interim analyses or stopping guidelines.

8a) CONSORT: Method used to generate the random allocation sequence

"Randomization was performed by a research member independent of data collection in a 1:1 ratio by a computerized program, TENALEA Clinical Trial Data Management System (NKI/AVL Biometrics department, Amsterdam, The Netherlands), using random block sizes (with maximum block size 6), stratified by study center."

8b) CONSORT: Type of randomisation; details of any restriction (such as blocking and block size)

"Randomization was performed by a research member independent of data collection in a 1:1 ratio by a computerized program, TENALEA Clinical Trial Data Management System (NKI/AVL Biometrics department, Amsterdam, The Netherlands), using random block sizes (with maximum block size 6), stratified by study center."

9) CONSORT: Mechanism used to implement the random allocation sequence (such as sequentially numbered containers), describing any steps taken to conceal the sequence until interventions were assigned

"Randomization was performed by a research member independent of data collection in a 1:1 ratio by a computerized program, TENALEA Clinical Trial Data Management System (NKI/AVL Biometrics department, Amsterdam, The Netherlands), using random block sizes (with maximum block size 6), stratified by study center."

10) CONSORT: Who generated the random allocation sequence, who enrolled participants, and who assigned participants to interventions

"Randomization was performed by a research member independent of data collection"

11a) CONSORT: Blinding - If done, who was blinded after assignment to interventions (for example, participants, care providers, those assessing outcomes) and how

11a-i) Specify who was blinded, and who wasn't

"Patients were asked not to share information about the randomization to the assessors, to ensure that they were blind to the allocated interventions."

11a-ii) Discuss e.g., whether participants knew which intervention was the "intervention of interest" and which one was the "comparator"

NA, there was only one intervention and one control group without intervention.

11b) CONSORT: If relevant, description of the similarity of interventions

NA

12a) CONSORT: Statistical methods used to compare groups for primary and secondary outcomes

"The effects of time of measurement, group and the group-by-time interaction were analyzed with linear mixed models."

12a-i) Imputation techniques to deal with attrition / missing values

"Missing data were imputed using general purpose multivariate imputation procedure (ICE: sequential regression imputation method), creating 50 different data sets. All analyses were performed using these 50 data sets and then pooled by combining the individual results."

12b) CONSORT: Methods for additional analyses, such as subgroup analyses and adjusted analyses

"Finally, as a post-hoc analysis, we applied latent growth mixture modelling (LGMM) [32,33] to explore possible latent subgroups within the 2 groups by use of the software Mplus (Version 6.11) [34] using a Bayesian estimator [35,36]."

RESULTS

13a) CONSORT: For each group, the numbers of participants who were randomly assigned, received intended treatment, and were analysed for the primary outcome

Figure 1 is a CONSORT flow diagram which contains the number of participants randomized, the attrition per group and the number of participants analysed for the primary outcome.

13b) CONSORT: For each group, losses and exclusions after randomisation, together with reasons

Flow chart figure 1 contains detailed information on losses and exclusions.

13b-i) Attrition diagram

Included in figure 1.

14a) CONSORT: Dates defining the periods of recruitment and follow-up

"Recruitment and follow-up took place from September 2007 to June 2010."

14a-i) Indicate if critical "secular events" fell into the study period

NA

14b) CONSORT: Why the trial ended or was stopped (early)

NA

15) CONSORT: A table showing baseline demographic and clinical characteristics for each group

Yes, table 1 is a baseline characteristics comparison.

15-i) Report demographics associated with digital divide issues

Basic information on age, gender, education, employment is provided in table 1.

16a) CONSORT: For each group, number of participants (denominator) included in each analysis and whether the analysis was by original assigned groups

16-i) Report multiple "denominators" and provide definitions

All analyses are performed on the original N's, as displayed in table 2. Completer analyses are reported separately.

16-ii) Primary analysis should be intent-to-treat

Yes, both requirements are met.

17a) CONSORT: For each primary and secondary outcome, results for each group, and the estimated effect size and its precision (such as 95% confidence interval)

Table 2 provides estimated means and 95% confidence intervals for all primary and secondary outcomes.

17a-i) Presentation of process outcomes such as metrics of use and intensity of use

"Intervention Usage
Most intervention group patients logged in on the intervention's website once (n=63, 41.7%). Fifty-four patients (35.8%) logged in multiple times (M=3.6, SD=3.5, Mdn=3, IQR=2-4). Thirty-four patients (22.5%) did not log in (e.g., non-users) and provided the following reasons: not interested anymore (2); occupied with rehabilitation (1); too busy (1); on holiday (1); too much on my mind (1); tired (1); difficulty concentrating (1); post-concussion symptoms (1); broken back (1); husband deceased (1); or no explanation (22). The average number of logins for the entire group was 1.7 (SD=2.5). The average login time was 20.8 minutes (SD=26.3). There were no differences in attrition or outcome measures between non-users (n=34) and users of the intervention (n=117), or between patients with a single login (n=63) versus multiple logins (n=54). The only differences were that more non-users than users had a non-Dutch cultural background (P=.003), and that patients with multiple logins were significantly older (mean age=48.0, SD=14.6) than those with a single login (mean age=39.6, SD=14.1; P=.001)."

17b) CONSORT: For binary outcomes, presentation of both absolute and relative effect sizes is recommended

NA

18) CONSORT: Results of any other analyses performed, including subgroup analyses and adjusted analyses, distinguishing pre-specified from exploratory

"Latent subgroups
Post-hoc LGMM analyses of self-reported PTSD symptoms (IES-R) revealed 2 latent subgroups per study group based on PTSD symptom severity at baseline, resulting in a low symptomatic control subgroup (n=94) and intervention subgroup (n=105) and a high symptomatic control subgroup (n=15) and intervention subgroup (n=20). The main difference between the groups was the slope of the high symptomatic subgroups, which showed a significant decrease in the intervention subgroup (P<.001), but not in the control subgroup (P=.32). Table 3 shows the outcomes of the LGMM analyses."

18-i) Subgroup analysis of comparing only users

"Completer analyses
In completers-only analyses (N=117 intervention group and N=149 control group patients), excluding non-users (n=34), results were similar to the intention-to-treat results for all outcome measures."

19) CONSORT: All important harms or unintended effects in each group

NA

19-i) Include privacy breaches, technical problems

NA

19-ii) Include qualitative feedback from participants or observations from staff/researchers

NA

DISCUSSION

20) CONSORT: Trial limitations, addressing sources of potential bias, imprecision, multiplicity of analyses

20-i) Typical limitations in ehealth trials

"As in comparable internet-based interventions [37], this study encountered non-utilization of the intervention, with one in five patients in the intervention group lacking any exposure to the intervention. In part, this non-usage was a consequence of a deliberate design choice, learning from debriefing research, allowing patients freedom in performing the intervention and choosing the content to increase their sense of control. A more strict approach to intervention adherence (e.g., a minimal number of logins or login time) could have resulted in greater benefits, though we found no differences in outcomes when comparing usage. These results should be interpreted cautiously, as our study was not adequately powered to find differences based on individual usage. Our less strict adherence approach, however, enhances external validity of the results, as uptake of the intervention by injury patients in daily clinical practice would not be 100%."

21) CONSORT: Generalisability (external validity, applicability) of the trial findings

21-i) Generalizability to other populations

"We do not know to what extent attrition may have biased our results, although besides marital status, we found no differences between participants and dropouts. In addition, our sample may not have been fully representative of the entire level-1 trauma center population, since we excluded patients with moderate-severe TBI, who did not master the Dutch language or who were unable to meet our time requirements for logging in."

21-ii) Discuss if there were elements in the RCT that would be different in a routine application setting

Since the main results of the trial were negative, a discussion on whether elements from the RCT would be different in a routine setting appears less useful.

22) CONSORT: Interpretation consistent with results, balancing benefits and harms, and considering other relevant evidence

22-i) Restate study questions and summarize the answers suggested by the data, starting with primary outcomes and process outcomes (use)

"In this paper, we presented the results of a randomized clinical trial comparing a self-guided internet-based prevention program versus usual care in the prevention of PTSD symptoms in injury patients.
PTSD symptoms decreased over time without a significant difference between the internet intervention group and the control group. In addition, we found no group differences on the number of patients diagnosed with PTSD or MDD or on depressive and anxiety symptoms at 12 months. Based on these results, there are currently no indications that offering a voluntary, information-based prevention program via the internet to unselected injury victims is useful in preventing PTSD symptoms."

22-ii) Highlight unanswered new questions, suggest future research

"In conclusion, our study found no evidence for preventing the development of PTSD symptoms by offering a voluntary, information-based prevention program via the internet to unselected injury trauma victims. Future research should determine the efficacy of applying interventions to selected individuals and should focus on increasing intervention usage."

Other information

23) CONSORT: Registration number and name of trial registry

"Trial number. www.controlled-trials.com Identifier: ISRCTN57754429"

24) CONSORT: Where the full trial protocol can be accessed, if available

"The full design and content of the intervention are described elsewhere [19,20]."

25) CONSORT: Sources of funding and other support (such as supply of drugs), role of funders

"Funding for this study was provided by the Netherlands Organization for Health Research and Development (ZonMw), Grant no. 62300038, and by the Achmea Foundation for Victims and Society (Stichting Achmea Slachtoffer en Samenleving). The role of the funders in the study was limited to providing financial support to conduct the trial and to oversee the general progress of the trial."

X26-i) Comment on ethics committee approval
"The local institutional review boards provided medical ethical approval."
x26-ii) Outline informed consent procedures
"Informed consent was obtained face-to-face directly prior to the baseline assessment at approximately 1 week post-injury."
X26-iii) Safety and security procedures
The intervention contained "contact information for program assistance or professional help for enduring symptoms".
X27-i) State the relation of the study team towards the system being evaluated
"Trauma TIPS (www.traumatips.nl, see Multimedia appendix 1) was created and is owned by the authors from the Research Group Psychotrauma [19]."
