# Supplementary figures and images for: Internet-Based Early Intervention to Prevent Posttraumatic Stress Disorder in Injury Patients: Randomized Controlled Trial
Source: J Med Internet Res. 2013 Aug 13;15(8):e165. doi: 10.2196/jmir.2460 (PMC3742408; doi:10.2196/jmir.2460)

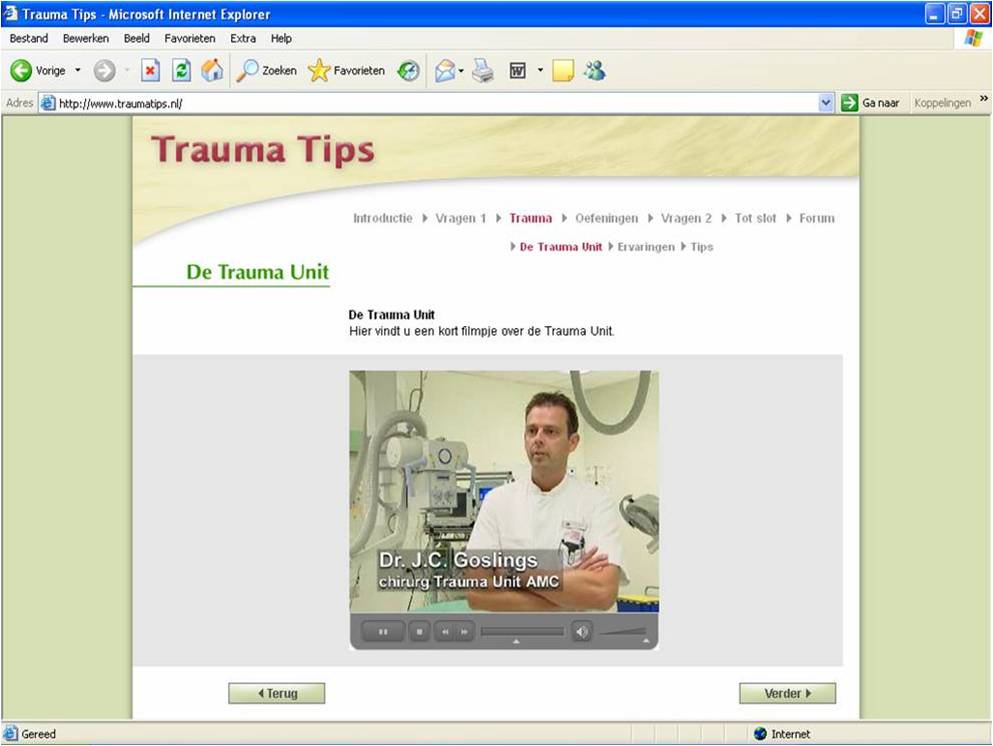

Supplement: Supplementary file 2 [file jmir_v15i8e165_app2.jpg]

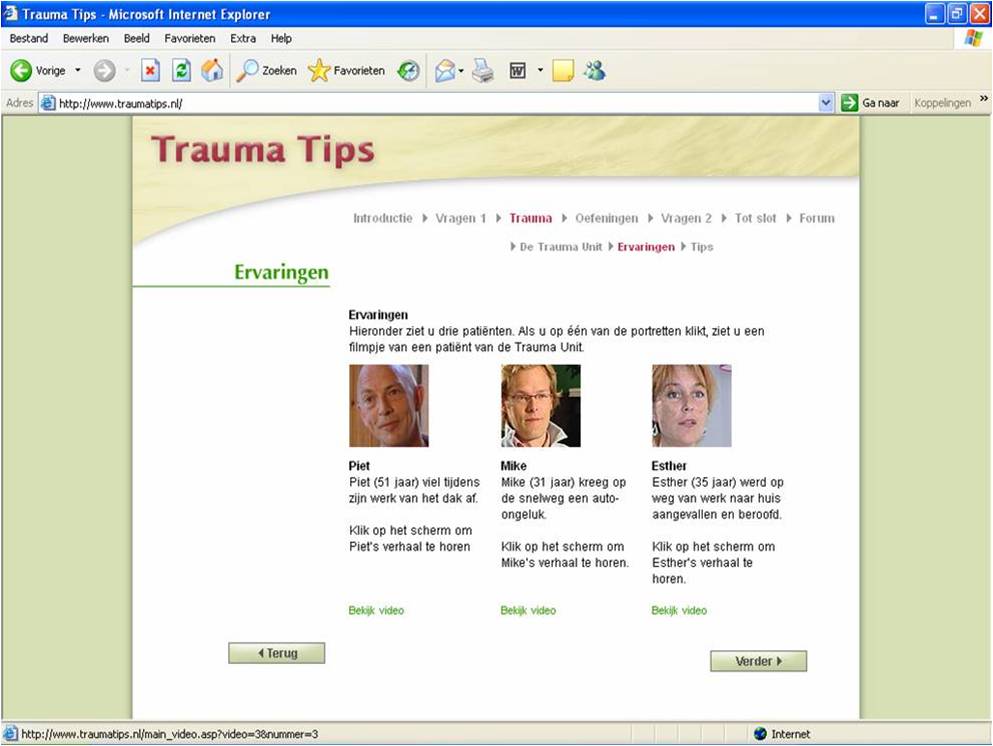

Supplement: Supplementary file 3 [file jmir_v15i8e165_app3.jpg]
